# Patient and/or family controlled palliative sedation with midazolam for intractable symptom control: a case series

**DOI:** 10.1186/1757-1626-2-136

**Published:** 2009-02-11

**Authors:** Diamanto Aretha, Eleftheria S Panteli, Panagiotis Kiekkas, Menelaos Karanikolas

**Affiliations:** 1 Department of Anaesthesiology and Critical Care Medicine, Patras University Hospital, Patras, Greece; 2 School of Medicine, Department of Anaesthesiology and Critical Care Medicine, University of Patras, Patras, Greece

## Abstract

**Introduction:**

Our case series prospectively evaluate the concept of Patient/Family-Controlled Sedation with midazolam, as an alternative to sedation by continuous infusion in terminal cancer patients.

**Cases presentation:**

Our method was applied in 8 pts. Midazolam was administered in a Patient Control Analgesia mode. The infusion pump was activated "as-needed" by the pt or a caretaker. Sedation was rated as: 1) awake 2) arousable to voice 3) arousable to light pain or 4) unarousable. Family satisfaction was rated as: 1) good, 2) fair, 3) poor, or 4) unacceptable. Mean midazolam consumption was 12 – 40 mg/24 hours. We did not observe respiratory depression. Death occurred 1–6 days after sedation started. Family satisfaction was mainly good and median sedation was in the range 2 – 3.

**Conclusion:**

Patient/Family-Controlled Sedation with midazolam was effective in providing comfort, by allowing titration of sedation to each patient's needs.

## Introduction

Relief of pain and suffering in patients with terminal illness is the main goal of Palliative Care [1-3]. However, despite state-of-the-art care, some patients continue to experience distressing end-of-life symptoms, including pain, dyspnea, anxiety, agitation and/or delirium. Palliative sedation is sometimes used in terminal patients with intractable symptoms, but its use has also been questioned on ethical grounds [4]. Palliative sedation use varies in different countries, and reported frequency is between 15% and 52% [5,1,8]. Provision of heavy sedation can cause significant distress to families because of concerns about sedation hastening death, and also because sedation often results in unconsciousness, depriving families from the opportunity to communicate with their loved ones [9,7,10]. There seems however to be a general understanding among palliative care practitioners that palliative or terminal sedation is used for intractable symptoms near the end of life [1]. The use of pharmacological agent(s) which could cause unconsciousness is truly distressing but causing death is not the intent. Of course, it may not be possible to achieve adequate symptom control except at the risk of shortening life (double effect) [11]. During the family discussion it is of paramount importance to make it clear that the main objective of palliative sedation is symptom relief and that this process can be established gradually and monitored carefully. Patient or/and Family-controlled sedation with midazolam in a PCA mode has not, to our knowledge, been described before. This pilot study is an attempt to evaluate the concept of Patient/Family Controlled Sedation (PFCS) with a single sedative (midazolam), as a novel, hopefully better alternative to sedation by continuous infusion in terminal patients with intractable distressing symptoms.

## Case presentation

The protocol was established in a Tertiary Care University Hospital. Data collection was prospective. All our patients were hospitalized in internal or pulmonary medicine wards and not in a special palliative care unit, as there are not such units in our country.

### Patients

Eight patients enrolled. In this case series the administration of high opioid doses had caused toxic side effects and this was the main inclusion criterion for our patients. All our patients suffered severe pain (refractory to systemic opioids, adjuvant analgesics, neurolytic procedures and/or epidural analgesia). Other inclusion criteria were distressing intractable symptoms (dyspnea, anxiety and agitation), age over 18, written patient or family consent, continuous availability of a competent adult at bedside, and very limited life expectancy, based on a palliative prognostic index score ≥ 8 [12]. Pain was assessed with VAS score, and was considered severe when VAS > 70. Dyspnea severity was measured with a numeric rating scale (NRS), and was considered mild when NRS = 1–3, median when NRS = 4–7 and severe when NRS > 7, on a 0–10 scale. NRS = 0 was considered as no dyspnea [13]. Agitation was assessed with the Richmond Agitation Sedation Scale (RASS) [14], and a patient was considered restless when RASS was +1, agitated and very agitated when RASS was +2 and +3 respectively and combative when RASS was +4. All patients had at least one symptom in addition to severe pain. At the time of evaluation by the Palliative Care Team all the patients were agitated or very agitated and three patients had moderate dyspnea (table 1).

**Table 1:** Patient characteristics and outcome

Case	Age/Sex	TUMOR (METASTASIS)	MAIN Symptoms	PPI	Family satisfaction	MEAN midazolam dose/day	analgesic dose/Day at the time sedation started
1	38, M	Gastric (lung, bowel)	pain, dyspnea, agitation	9	good	17 mg	TTS Fentanyl 100 mcg/h + Morphine 80 mg/day IV
2	24, M	Kidney (lung, omentum)	pain, dyspnea, agitation	10	good	34 mg	TTS Fentanyl 75 mcg/h + Morphine 100 mg/day IV
3	64, F	Colon (bone, bowel, pelvis, rectum)	pain, agitation	9	good	12 mg	TTS Fentanyl 75 mcg/h + Morphine 40 mg/day IV
4	55, M	Pancreas (liver, bowel)	pain, dyspnea, agitation	9	fair	20 mg	TTS Fentanyl 150 mcg/h + Morphine 60 mg/day IV
5	73, F	Pancreas (bowel, liver)	pain, agitation	10	good	15 mg	TTS Fentanyl 50 mcg/h + Morphine 30 mg/day IV
6	40, M	Gastric (liver, lung)	Pain, agitation	8	good	40 mg	TTS Fentanyl 150 mcg/h + Morphine 40 mg/day IV
7	64, F	Liver (Lung)	Pain, agitation	8	good	25 mg	TTS Fentanyl 150 mcg/h + Epidural analgesia (Levobupivacaine 2,2 mg/ml, Fentanyl 10 mcg/ml, 5–10 ml/h)
8	67, M	Liver (bowel, lung)	Pain, agitation	9	good	19 mg	TTS Fentanyl 100 mcg/h + Epidural analgesia (Levobupivacaine 2 mg/ml, Fentanyl 5 mcg/ml, 5–9 ml/h)

### Palliative sedation protocol

Midazolam was administered intravenously by a programmable electronic pump (Abbott laboratories, Inc, Gemstar^®^) in a PCA (Patient Control Analgesia) mode. Initial settings were bolus 0.3 mg, lockout 20 minutes and no basal infusion, and dose was adjusted to patient comfort. The PFCS protocol allowed pump activation on an "as-needed" basis by either the patient or a first degree relative, depending on patient's condition. A physician evaluated each patient, at least, four times daily, recorded sedation scale, respiratory rate, dyspnea NRS, RASS, VAS pain score and family satisfaction and adjusted the pump as needed. Sedation was rated as: 1) awake 2) arousable with voice 3) arousable with light pain and 4) unarousable. Family satisfaction was rated by the family member as: 1) good, 2) fair, 3) poor, or 4) unacceptable. Family caretakers were trained and instructed to observe the respiratory rate, and call a physician if the patient seemed agitated, in pain, or for respiratory rate less than 12/minute. PFCS was adjusted as needed to achieve a balance between comfort and sedation.

## Results

PFCS was applied on 8 patients in a four years period. Mean midazolam consumption was between 12 mg/day (case 3) and 40 mg/day (case 6, table 1). All patients continued their systemic opoid analgesic regimen (iv morphine, TTS Fentanyl), which was gradually reduced from the maximal dose, or/and their epidural infusion. We did not observe respiratory depression in any patient, and respiratory distress, when present, improved. In one patient (case 8) RR/min decreased seriously from 20 to 8 (three days after PFCS with midazolam started) and we lightened the midazolam dose. NRS decreased from 7 to 5 in cases 1 and 2 and from 7 to 4 in case 5. The sedation score in table 2 is the median daily sedation score – 4 daily measurements at 6 hours intervals. Death occurred 1–6 days after PFCS started (table 2). Family satisfaction was good in seven patients and fair in one patient (case 4), and median sedation score was in the range 2–3. In case 4, family members wanted the patient to be more awake but distressing symptoms recurred on awakening. Patient, treatment and outcome data, including family satisfaction, are summarized in table 1.

**Table 2:** Sedation scores

	Median Sedation					
	
Case	Day 1	Day 2	Day 3	Day 4	Day 5	Day 6
1	3	3	2	death		
2	3	1	death			
3	3	death				
4	3	3	3	death		
5	3	3	2	death		
6	1	2	2	3	3	death
7	2	2	2	3	2	3
8	1	2	3	3	4	3

## Discussion

When provision of adequate end-of-life symptom control becomes challenging, sedation can be a very useful therapeutic tool [15]. In such cases, sedation is applied with intent to provide comfort, but also with the understanding that it could hasten death [16,7]. Our case series have many limitations. Management of the infusion by health care providers could also allow for titration of the dose and perhaps be safer. On the other hand, in my country terminal cancer patients are usually hospitalized in common medical wards, not in a special palliative care unit, hospitals are understaffed and the care provided by family caretakers is very important. Physicians evaluated the patients many times daily (at least four) and were giving instructions, but the patient would not be safe without a continue availability of a family caretaker at bedside (which, unfortunately, is something very usual in understaffed hospitals). We had only 8 patients during a 4 years period. We were asked to "do something" only in "difficult cases". All our patients had refractory symptoms and high opioid doses, when had been used, caused toxic side effects. On the other hand midazolam doses were low and well titrated and our patients became calm. We could not use continuous midazolam infusions as in common medical wards continuous infusions could be very dangerous. Although the sedation protocol is called "patient-family control sedation", in reality patients were only able to activate the pump in the beginning of the PFCS protocol; subsequently the pump was activated mostly by family members. Our midazolam PFCS protocol was different compared to previous reports of end-of-life sedations in four important ways:

i. Data collection was prospective, and to our knowledge only one study was prospective [17].

ii. A single sedative (midazolam) was used

iii. Sedation was administered in a "PCA" mode, which allows for dose titration to the needs of each patient, and

iv. Family members could control drug use and lighten sedation when there was a need or desire to communicate with the patient. This "control" element significantly reduced family distress, as family members clearly understood that sedation was titrated to comfort, and what they provided to their loved-ones was sedation, not euthanasia [9].

Prospective research is difficult in this population, but symptom control, patient and family acceptance, and untoward effects should be the most important endpoints of a study. Several sedatives have been tried with midazolam being the most common. Choice of agent is primary based on efficacy and then on unwanted effects, available routes of administration and cost. Opioids are frequently used to treat pain and other symptoms. But these medications may be inadequate to control symptoms prior to death. This protocol has focused on nonopioids drugs. All the patients were receiving opioids and two patients epidural infusion in addition to the sedative employed for terminal sedation. There is a practice of using high doses of opioids for terminal sedation which did not happened with these 8 patients. Sometimes we consider this to be unwise as opioids are used in excess of that required to relieve dyspnea or pain. The use of a specific sedative agent to produce terminal sedation and comfort is much better. All our patients survived for more than one day. This survival is similar to others [8]. Survival for more than 1 day with high therapeutic efficacy suggests that terminal sedation did not cause premature death. We believe that the "PCA" component is very important in this PFCS protocol, because it allows titration of sedation to individual patient needs and may therefore explain the good response we observed in most patients.

## Conclusion

In our experience, PFCS with midazolam was effective in providing comfort, by allowing optimization of sedation to each patient's needs. PFCS may be an alternative to the standard continuous infusion technique for terminal sedation. However, this report is only a prospective data collection, not a prospective clinical trial, and includes a small number of patients. Therefore, prospective studies comparing the effectiveness of PFCS to that of continuous infusion regimens in more patients are needed to support or disprove the validity of our conclusions.

## Consent

Written informed consent was obtained retrospectively from the patient for publication of this case report and accompanying images. A copy of the written consent is available for review by the Editor-in-Chief of this journal.

## Competing interests

The authors declare that they have no competing interests.

## Authors' contributions

DA participated in the design of the study, in the data collection in the analysis of the data and prepared the manuscript. MK participated in the design of the study, in the data collection in the analysis of the data, reviewed and revised the manuscript. ESP participated in the data collection and in the analysis of the data. PK participated in the analysis of the data and revised the manuscript.
